# {6,6′-Dimeth­oxy-2,2′-[ethane-1,2-diylbis(nitrilo­methanylyl­idene)]diphenolato}nickel(II) dimethyl­formamide monosolvate

**DOI:** 10.1107/S1600536811004818

**Published:** 2011-02-12

**Authors:** Kouassi Ayikoé, Ray J. Butcher, Yilma Gultneh

**Affiliations:** a Department of Chemistry, Howard University, 525 College Street NW, Washington, DC 20059, USA

## Abstract

In the title compound, [Ni(C_18_H_18_N_2_O_4_)]·C_3_H_7_NO, the central Ni^II^ atom is in a square-planar O_2_N_2_ coordination environment. The planar Ni–salen moieties (r.m.s. deviation for the plane through the conjugated part of the Ni–salen group = 0.07 Å) form parallel stacks in the *a*-axis direction, with alternating Ni⋯Ni separations of 3.5339 (7) and 3.6165 (7) Å. In the crystal, there are weak inter­molecular C—H⋯O inter­actions involving the dimethyl­formamide O and phenolate O atoms.

## Related literature

For stacking of Ni–salen units, see: Abe *et al.* (2006 ▶); Assey *et al.* (2010 ▶); Feng *et al.* (2007 ▶); Miyamura *et al.* (1995 ▶); Vasil’eva *et al.* (2003 ▶). For a description of the Cambridge Structural Database, see: Allen (2002 ▶).
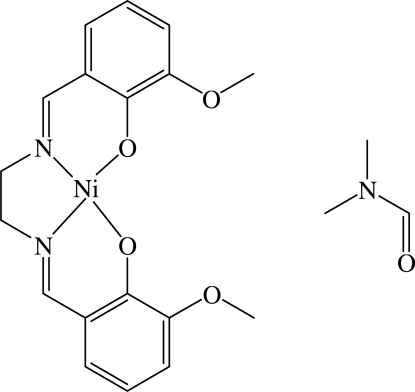


## Experimental

### 

#### Crystal data

[Ni(C_18_H_18_N_2_O_4_)]·C_3_H_7_NO
*M* *_r_* = 458.15
Monoclinic, 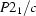

*a* = 6.8601 (1) Å
*b* = 15.3432 (3) Å
*c* = 18.9065 (4) Å
β = 91.676 (2)°
*V* = 1989.17 (6) Å^3^
*Z* = 4
Cu *K*α radiation
μ = 1.75 mm^−1^
*T* = 110 K
0.53 × 0.35 × 0.28 mm

#### Data collection

Oxford Diffraction Xcalibur diffractometer with a Ruby detector
Absorption correction: multi-scan (*CrysAlis RED*; Oxford Diffraction, 2009 ▶) *T*_min_ = 0.750, *T*_max_ = 1.000
7909 measured reflections
3911 independent reflections
3513 reflections with *I* > 2σ(*I*)
*R*_int_ = 0.020

#### Refinement

*R*[*F* ^2^ > 2σ(*F* ^2^)] = 0.041
*wR*(*F* ^2^) = 0.114
*S* = 1.10
3911 reflections
275 parameters
H-atom parameters constrained
Δρ_max_ = 0.34 e Å^−3^
Δρ_min_ = −0.31 e Å^−3^

### 

Data collection: *CrysAlis PRO* (Oxford Diffraction, 2009 ▶); cell refinement: *CrysAlis PRO*; data reduction: *CrysAlis PRO*; program(s) used to solve structure: *SHELXS97* (Sheldrick, 2008 ▶); program(s) used to refine structure: *SHELXL97* (Sheldrick, 2008 ▶); molecular graphics: *SHELXTL* (Sheldrick, 2008 ▶); software used to prepare material for publication: *SHELXTL*.

## Supplementary Material

Crystal structure: contains datablocks I, global. DOI: 10.1107/S1600536811004818/tk2717sup1.cif

Structure factors: contains datablocks I. DOI: 10.1107/S1600536811004818/tk2717Isup2.hkl

Additional supplementary materials:  crystallographic information; 3D view; checkCIF report

## Figures and Tables

**Table 1:** Selected bond lengths (Å)

Ni—N1	1.8503 (17)
Ni—N2	1.8502 (17)
Ni—O1	1.8609 (13)
Ni—O2	1.8594 (13)

**Table 2:** Hydrogen-bond geometry (Å, °)

*D*—H⋯*A*	*D*—H	H⋯*A*	*D*⋯*A*	*D*—H⋯*A*
C4—H4*A*⋯O1*S*	0.95	2.62	3.310 (3)	130
C9—H9*A*⋯O2^i^	0.99	2.45	3.334 (3)	148
C10—H10*A*⋯O1^ii^	0.99	2.45	3.348 (3)	151

Symmetry codes: (i) 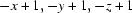; (ii) 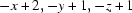.

**Fig. 1.**
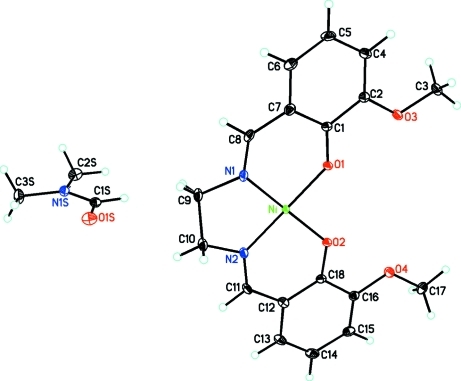
The molecular structures of the complex, C18H18N3NiO5, and solvent, C3H7NO, showing the atom numbering scheme and 50% probability displacement ellipsoids.

**Fig. 2.**
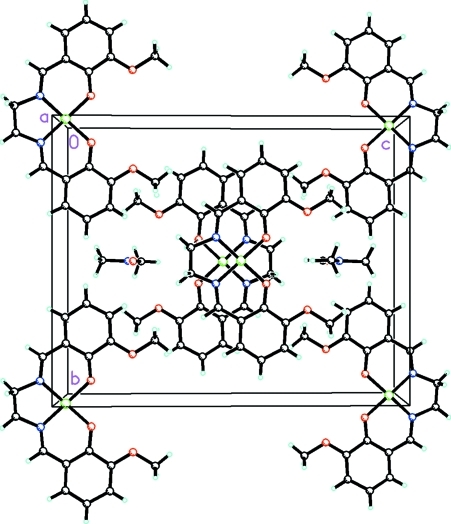
The molecular packing for C18H18N3NiO5.C3H7NO, viewed down the a axis showing overlapping Nisalen units.

## supplementary crystallographic information

### Comment

The title compound is a composed of one mononuclear nickel salen-type complex and one molecule of dimethylformamide as solvate. The central Ni is in a square planar O_2_N_2_ coordination environment (Fig. 1). The Ni—N and Ni—O bond distances, Table 1, are in the normal range for Ni-salen type complexes (Allen, 2002). The planar Ni salen moieties form parallel stacks in the *a* direction with alternating Ni—Ni separations of 3.5339 (7) and 3.6165 (7) Å as is common for this type of complex (Abe *et al.*, 2006; Assey *et al.*, 2010; Feng *et al.*, 2007; Miyamura *et al.*, 1995; Vasil'eva *et al.*, 2003). There are weak intermolecular C—H···O interactions involving the DMF O and phenolic O atoms (Table 2 and Fig. 2).

### Experimental

The ligand, *N*,*N*'-bis(3-methoxysalicylaldehyde)ethylenediimine (H_2_*L*_1_), was synthesized in a conventional way by mixing *o*-vanillin with ethylenediamine. To 10 g (0.066 mole) of *o*-vanillin weighed into a 100 ml round-bottom flask, 30 ml of methanol and 3 mL (0.035 mole) of ethylenediamine were added drop-wise while stirring. The resulting mixture was refluxed at a regulated temperature of 313 K overnight. The yellow precipitate was filtered under vacuum, dissolved in methanol and filtered a second time.

To a stirred bright yellow solution of 20 ml methanol containing 0.9 g (2.74 mmol) of H_2_*L*_1_, a 20 ml green methanol solution of 0.65 g NiCl_2_.6H_2_O (2.73 mmol) was added drop-wise with continuous stirring. About 2 to 3 drops of triethylamine was added to activate deprotonation of the 2-hydroxyl group on the aldehyde moiety and promote oxygen binding to the metal. The resulting dark brown complex solution was stirred and refluxed overnight at 313 K, rotary-evaporated and washed with ethanol to obtain a brown solid in over 90% yield. The complex (7 mg) was dissolved in 5 ml of *N*,*N*'-dimethyl formamide and filtered into a crystallization tube. Sufficient amount of diethyl ether was slowly layered over the dissolved complex yielding red brown crystals after several days.

### Refinement

H atoms were placed in geometrically idealized positions and constrained to ride on their parent atoms with C—H distances of 0.95 to 0.99 Å and *U*_iso_(H) = 1.2*U*_eq_(C) [*U*_iso_(H) = 1.5*U*_eq_(C) for CH_3_].

### Crystal data

**Table:**

[Ni(C_18_H_18_N_2_O_4_)]·C_3_H_7_NO	*F*(000) = 960
*M**_r_* = 458.15	*D*_x_ = 1.530 Mg m^−^^3^
Monoclinic, *P*2_1_/*c*	Cu *K*α radiation, λ = 1.54178 Å
Hall symbol: -P 2ybc	Cell parameters from 5823 reflections
*a* = 6.8601 (1) Å	θ = 5.5–73.9°
*b* = 15.3432 (3) Å	µ = 1.75 mm^−^^1^
*c* = 18.9065 (4) Å	*T* = 110 K
β = 91.676 (2)°	Prism, red brown
*V* = 1989.17 (6) Å^3^	0.53 × 0.35 × 0.28 mm
*Z* = 4	

### Data collection

**Table:**

Oxford Diffraction Xcalibur diffractometer with a Ruby (Gemini Mo) detector	3911 independent reflections
Radiation source: Enhance (Cu) X-ray Source	3513 reflections with *I* > 2σ(*I*)
graphite	*R*_int_ = 0.020
Detector resolution: 10.5081 pixels mm^-1^	θ_max_ = 74.1°, θ_min_ = 5.5°
ω scans	*h* = −8→6
Absorption correction: multi-scan (*CrysAlis RED*; Oxford Diffraction, 2009)	*k* = −18→18
*T*_min_ = 0.750, *T*_max_ = 1.000	*l* = −22→23
7909 measured reflections	

### Refinement

**Table:**

Refinement on *F*^2^	Primary atom site location: structure-invariant direct methods
Least-squares matrix: full	Secondary atom site location: difference Fourier map
*R*[*F*^2^ > 2σ(*F*^2^)] = 0.041	Hydrogen site location: inferred from neighbouring sites
*wR*(*F*^2^) = 0.114	H-atom parameters constrained
*S* = 1.10	*w* = 1/[σ^2^(*F*_o_^2^) + (0.0443*P*)^2^ + 2.5615*P*] where *P* = (*F*_o_^2^ + 2*F*_c_^2^)/3
3911 reflections	(Δ/σ)_max_ = 0.001
275 parameters	Δρ_max_ = 0.34 e Å^−^^3^
0 restraints	Δρ_min_ = −0.31 e Å^−^^3^

### Special details

**Table:**

Geometry. All e.s.d.'s (except the e.s.d. in the dihedral angle between two l.s. planes) are estimated using the full covariance matrix. The cell e.s.d.'s are taken into account individually in the estimation of e.s.d.'s in distances, angles and torsion angles; correlations between e.s.d.'s in cell parameters are only used when they are defined by crystal symmetry. An approximate (isotropic) treatment of cell e.s.d.'s is used for estimating e.s.d.'s involving l.s. planes.
Refinement. Refinement of *F*^2^ against ALL reflections. The weighted *R*-factor *wR* and goodness of fit *S* are based on *F*^2^, conventional *R*-factors *R* are based on *F*, with *F* set to zero for negative *F*^2^. The threshold expression of *F*^2^ > σ(*F*^2^) is used only for calculating *R*-factors(gt) *etc*. and is not relevant to the choice of reflections for refinement. *R*-factors based on *F*^2^ are statistically about twice as large as those based on *F*, and *R*- factors based on ALL data will be even larger.

### Fractional atomic coordinates and isotropic or equivalent isotropic displacement parameters (Å^2^)

**Table:**

	*x*	*y*	*z*	*U*_iso_*/*U*_eq_	
Ni	0.74901 (5)	0.498467 (19)	0.526650 (16)	0.01230 (13)	
O1	0.7304 (2)	0.41708 (9)	0.59911 (7)	0.0156 (3)	
O2	0.7642 (2)	0.58041 (9)	0.59906 (7)	0.0153 (3)	
O3	0.7206 (2)	0.32568 (9)	0.71759 (7)	0.0202 (3)	
O4	0.7569 (2)	0.67226 (9)	0.71712 (7)	0.0219 (3)	
O1S	0.5907 (2)	−0.00040 (9)	0.71449 (9)	0.0270 (4)	
N1	0.7406 (2)	0.41614 (11)	0.45511 (9)	0.0159 (3)	
N2	0.7631 (2)	0.57987 (11)	0.45478 (9)	0.0160 (3)	
N1S	0.2629 (3)	−0.00026 (10)	0.69058 (10)	0.0191 (4)	
C1	0.7385 (3)	0.33191 (13)	0.59320 (10)	0.0143 (4)	
C2	0.7330 (3)	0.27939 (13)	0.65578 (10)	0.0159 (4)	
C3	0.7344 (3)	0.27729 (14)	0.78197 (11)	0.0214 (4)	
H3A	0.7272	0.3173	0.8222	0.032*	
H3B	0.8588	0.2459	0.7845	0.032*	
H3C	0.6266	0.2355	0.7834	0.032*	
C4	0.7376 (3)	0.18962 (13)	0.65251 (11)	0.0198 (4)	
H4A	0.7304	0.1564	0.6948	0.024*	
C5	0.7531 (3)	0.14675 (13)	0.58720 (12)	0.0257 (5)	
H5A	0.7577	0.0849	0.5853	0.031*	
C6	0.7613 (3)	0.19459 (15)	0.52644 (12)	0.0251 (5)	
H6A	0.7735	0.1657	0.4823	0.030*	
C7	0.7518 (3)	0.28656 (14)	0.52840 (11)	0.0191 (4)	
C8	0.7480 (3)	0.33263 (14)	0.46263 (11)	0.0194 (4)	
H8A	0.7513	0.2987	0.4207	0.023*	
C9	0.7123 (3)	0.45208 (15)	0.38295 (10)	0.0219 (4)	
H9A	0.5720	0.4523	0.3692	0.026*	
H9B	0.7824	0.4159	0.3486	0.026*	
C10	0.7908 (3)	0.54324 (14)	0.38323 (10)	0.0215 (4)	
H10A	0.9310	0.5429	0.3722	0.026*	
H10B	0.7202	0.5789	0.3472	0.026*	
C11	0.7590 (3)	0.66365 (14)	0.46181 (11)	0.0195 (4)	
H11A	0.7622	0.6974	0.4197	0.023*	
C12	0.7500 (3)	0.71005 (14)	0.52714 (11)	0.0192 (4)	
C13	0.7388 (3)	0.80217 (15)	0.52450 (12)	0.0252 (5)	
H13A	0.7355	0.8308	0.4799	0.030*	
C14	0.7328 (3)	0.85024 (14)	0.58504 (13)	0.0268 (5)	
H14A	0.7254	0.9120	0.5827	0.032*	
C15	0.7376 (3)	0.80773 (13)	0.65102 (12)	0.0203 (4)	
H15A	0.7318	0.8411	0.6933	0.024*	
C16	0.7506 (3)	0.71821 (13)	0.65496 (10)	0.0164 (4)	
C17	0.7597 (3)	0.72153 (15)	0.78123 (11)	0.0220 (4)	
H17A	0.7687	0.6818	0.8218	0.033*	
H17B	0.6397	0.7559	0.7836	0.033*	
H17C	0.8726	0.7607	0.7823	0.033*	
C18	0.7554 (3)	0.66541 (13)	0.59253 (10)	0.0146 (4)	
C1S	0.4214 (3)	−0.00156 (11)	0.73382 (12)	0.0181 (4)	
H1SA	0.4011	−0.0035	0.7833	0.022*	
C2S	0.0692 (3)	−0.00288 (14)	0.71929 (14)	0.0270 (5)	
H2SA	0.0795	−0.0017	0.7711	0.040*	
H2SB	0.0029	−0.0564	0.7038	0.040*	
H2SC	−0.0057	0.0478	0.7024	0.040*	
C3S	0.2806 (5)	0.00255 (16)	0.61430 (13)	0.0347 (6)	
H3SA	0.4173	0.0120	0.6028	0.052*	
H3SB	0.2009	0.0503	0.5948	0.052*	
H3SC	0.2355	−0.0528	0.5938	0.052*	

### Atomic displacement parameters (Å^2^)

**Table:**

	*U*^11^	*U*^22^	*U*^33^	*U*^12^	*U*^13^	*U*^23^
Ni	0.0174 (2)	0.0113 (2)	0.00815 (19)	−0.00089 (12)	0.00040 (13)	0.00026 (11)
O1	0.0232 (7)	0.0109 (6)	0.0127 (6)	−0.0014 (5)	0.0010 (5)	0.0000 (5)
O2	0.0216 (7)	0.0113 (6)	0.0131 (6)	−0.0013 (5)	0.0005 (5)	−0.0001 (5)
O3	0.0333 (8)	0.0145 (7)	0.0130 (7)	0.0003 (6)	0.0024 (6)	0.0026 (5)
O4	0.0373 (9)	0.0150 (7)	0.0135 (7)	−0.0009 (6)	0.0018 (6)	−0.0027 (5)
O1S	0.0217 (8)	0.0249 (9)	0.0347 (9)	0.0000 (6)	0.0039 (7)	0.0014 (6)
N1	0.0175 (8)	0.0195 (9)	0.0105 (8)	−0.0004 (7)	−0.0001 (6)	−0.0017 (6)
N2	0.0169 (8)	0.0201 (9)	0.0110 (8)	−0.0018 (6)	−0.0002 (6)	0.0023 (6)
N1S	0.0239 (9)	0.0172 (9)	0.0162 (9)	−0.0004 (7)	−0.0001 (7)	0.0003 (6)
C1	0.0135 (9)	0.0121 (9)	0.0173 (9)	−0.0003 (7)	0.0005 (7)	−0.0002 (7)
C2	0.0140 (9)	0.0163 (10)	0.0176 (9)	−0.0002 (7)	0.0012 (7)	0.0000 (7)
C3	0.0244 (11)	0.0227 (10)	0.0171 (10)	−0.0001 (8)	0.0008 (8)	0.0067 (8)
C4	0.0203 (10)	0.0152 (10)	0.0238 (11)	−0.0002 (8)	−0.0001 (8)	0.0035 (8)
C5	0.0328 (12)	0.0108 (9)	0.0334 (13)	−0.0007 (8)	−0.0017 (10)	−0.0022 (8)
C6	0.0339 (12)	0.0177 (11)	0.0237 (11)	−0.0002 (9)	0.0001 (9)	−0.0073 (8)
C7	0.0207 (10)	0.0175 (10)	0.0190 (10)	0.0012 (8)	−0.0008 (8)	−0.0036 (8)
C8	0.0233 (10)	0.0202 (10)	0.0146 (9)	−0.0008 (8)	0.0001 (8)	−0.0074 (8)
C9	0.0272 (11)	0.0281 (11)	0.0104 (9)	−0.0022 (9)	−0.0006 (8)	−0.0006 (8)
C10	0.0259 (11)	0.0274 (11)	0.0114 (9)	−0.0025 (9)	0.0018 (8)	0.0014 (8)
C11	0.0235 (10)	0.0194 (10)	0.0155 (9)	−0.0012 (8)	−0.0005 (8)	0.0079 (8)
C12	0.0205 (10)	0.0175 (10)	0.0195 (10)	0.0008 (8)	0.0020 (8)	0.0034 (8)
C13	0.0320 (12)	0.0164 (10)	0.0272 (12)	−0.0003 (9)	−0.0001 (9)	0.0087 (8)
C14	0.0347 (13)	0.0111 (10)	0.0344 (13)	−0.0007 (9)	0.0005 (10)	0.0022 (9)
C15	0.0192 (10)	0.0146 (10)	0.0270 (11)	−0.0005 (8)	0.0018 (8)	−0.0043 (8)
C16	0.0137 (9)	0.0161 (9)	0.0192 (10)	−0.0008 (7)	−0.0002 (7)	0.0000 (8)
C17	0.0250 (11)	0.0232 (11)	0.0178 (10)	0.0007 (8)	0.0010 (8)	−0.0079 (8)
C18	0.0135 (9)	0.0124 (9)	0.0179 (10)	−0.0008 (7)	−0.0002 (7)	0.0002 (7)
C1S	0.0229 (10)	0.0114 (9)	0.0200 (10)	−0.0017 (7)	0.0006 (8)	−0.0005 (7)
C2S	0.0208 (11)	0.0254 (12)	0.0347 (13)	−0.0012 (8)	0.0004 (9)	0.0028 (9)
C3S	0.0537 (17)	0.0342 (14)	0.0161 (11)	0.0016 (11)	−0.0033 (11)	−0.0005 (9)

### Geometric parameters (Å, °)

**Table:**

Ni—N1	1.8503 (17)	C6—H6A	0.9500
Ni—N2	1.8502 (17)	C7—C8	1.430 (3)
Ni—O1	1.8609 (13)	C8—H8A	0.9500
Ni—O2	1.8594 (13)	C9—C10	1.499 (3)
O1—C1	1.313 (2)	C9—H9A	0.9900
O2—C18	1.311 (2)	C9—H9B	0.9900
O3—C2	1.372 (2)	C10—H10A	0.9900
O3—C3	1.427 (2)	C10—H10B	0.9900
O4—C16	1.370 (2)	C11—C12	1.429 (3)
O4—C17	1.428 (2)	C11—H11A	0.9500
O1S—C1S	1.228 (3)	C12—C18	1.413 (3)
N1—C8	1.290 (3)	C12—C13	1.416 (3)
N1—C9	1.479 (2)	C13—C14	1.363 (3)
N2—C11	1.293 (3)	C13—H13A	0.9500
N2—C10	1.482 (2)	C14—C15	1.407 (3)
N1S—C1S	1.341 (3)	C14—H14A	0.9500
N1S—C2S	1.451 (3)	C15—C16	1.378 (3)
N1S—C3S	1.451 (3)	C15—H15A	0.9500
C1—C7	1.414 (3)	C16—C18	1.433 (3)
C1—C2	1.433 (3)	C17—H17A	0.9800
C2—C4	1.379 (3)	C17—H17B	0.9800
C3—H3A	0.9800	C17—H17C	0.9800
C3—H3B	0.9800	C1S—H1SA	0.9500
C3—H3C	0.9800	C2S—H2SA	0.9800
C4—C5	1.406 (3)	C2S—H2SB	0.9800
C4—H4A	0.9500	C2S—H2SC	0.9800
C5—C6	1.366 (3)	C3S—H3SA	0.9800
C5—H5A	0.9500	C3S—H3SB	0.9800
C6—C7	1.413 (3)	C3S—H3SC	0.9800
			
N2—Ni—N1	85.71 (8)	C10—C9—H9B	110.1
N2—Ni—O2	94.66 (7)	H9A—C9—H9B	108.4
N1—Ni—O2	178.51 (7)	N2—C10—C9	107.53 (16)
N2—Ni—O1	179.01 (7)	N2—C10—H10A	110.2
N1—Ni—O1	94.51 (7)	C9—C10—H10A	110.2
O2—Ni—O1	85.14 (6)	N2—C10—H10B	110.2
C1—O1—Ni	126.96 (12)	C9—C10—H10B	110.2
C18—O2—Ni	127.00 (12)	H10A—C10—H10B	108.5
C2—O3—C3	116.94 (16)	N2—C11—C12	125.87 (19)
C16—O4—C17	117.06 (16)	N2—C11—H11A	117.1
C8—N1—C9	118.40 (17)	C12—C11—H11A	117.1
C8—N1—Ni	126.67 (15)	C18—C12—C13	120.97 (19)
C9—N1—Ni	114.85 (13)	C18—C12—C11	120.96 (19)
C11—N2—C10	118.33 (17)	C13—C12—C11	118.07 (19)
C11—N2—Ni	126.45 (14)	C14—C13—C12	120.9 (2)
C10—N2—Ni	115.16 (13)	C14—C13—H13A	119.6
C1S—N1S—C2S	120.44 (19)	C12—C13—H13A	119.6
C1S—N1S—C3S	121.1 (2)	C13—C14—C15	119.53 (19)
C2S—N1S—C3S	118.5 (2)	C13—C14—H14A	120.2
O1—C1—C7	124.57 (18)	C15—C14—H14A	120.2
O1—C1—C2	119.16 (17)	C16—C15—C14	120.6 (2)
C7—C1—C2	116.27 (18)	C16—C15—H15A	119.7
O3—C2—C4	123.86 (18)	C14—C15—H15A	119.7
O3—C2—C1	114.57 (17)	O4—C16—C15	124.02 (19)
C4—C2—C1	121.56 (18)	O4—C16—C18	114.50 (17)
O3—C3—H3A	109.5	C15—C16—C18	121.47 (19)
O3—C3—H3B	109.5	O4—C17—H17A	109.5
H3A—C3—H3B	109.5	O4—C17—H17B	109.5
O3—C3—H3C	109.5	H17A—C17—H17B	109.5
H3A—C3—H3C	109.5	O4—C17—H17C	109.5
H3B—C3—H3C	109.5	H17A—C17—H17C	109.5
C2—C4—C5	120.60 (19)	H17B—C17—H17C	109.5
C2—C4—H4A	119.7	O2—C18—C12	124.36 (18)
C5—C4—H4A	119.7	O2—C18—C16	119.15 (17)
C6—C5—C4	119.57 (19)	C12—C18—C16	116.49 (18)
C6—C5—H5A	120.2	O1S—C1S—N1S	125.1 (2)
C4—C5—H5A	120.2	O1S—C1S—H1SA	117.4
C5—C6—C7	120.8 (2)	N1S—C1S—H1SA	117.4
C5—C6—H6A	119.6	N1S—C2S—H2SA	109.5
C7—C6—H6A	119.6	N1S—C2S—H2SB	109.5
C6—C7—C1	121.21 (19)	H2SA—C2S—H2SB	109.5
C6—C7—C8	118.07 (19)	N1S—C2S—H2SC	109.5
C1—C7—C8	120.68 (19)	H2SA—C2S—H2SC	109.5
N1—C8—C7	125.91 (19)	H2SB—C2S—H2SC	109.5
N1—C8—H8A	117.0	N1S—C3S—H3SA	109.5
C7—C8—H8A	117.0	N1S—C3S—H3SB	109.5
N1—C9—C10	107.90 (16)	H3SA—C3S—H3SB	109.5
N1—C9—H9A	110.1	N1S—C3S—H3SC	109.5
C10—C9—H9A	110.1	H3SA—C3S—H3SC	109.5
N1—C9—H9B	110.1	H3SB—C3S—H3SC	109.5
			
N2—Ni—O1—C1	111 (4)	C2—C1—C7—C8	176.74 (18)
N1—Ni—O1—C1	8.14 (16)	C9—N1—C8—C7	−172.29 (19)
O2—Ni—O1—C1	−170.41 (16)	Ni—N1—C8—C7	4.3 (3)
N2—Ni—O2—C18	8.98 (16)	C6—C7—C8—N1	−179.6 (2)
N1—Ni—O2—C18	113 (3)	C1—C7—C8—N1	2.8 (3)
O1—Ni—O2—C18	−170.05 (16)	C8—N1—C9—C10	−156.46 (19)
N2—Ni—N1—C8	172.71 (18)	Ni—N1—C9—C10	26.6 (2)
O2—Ni—N1—C8	68 (3)	C11—N2—C10—C9	−157.70 (19)
O1—Ni—N1—C8	−8.25 (18)	Ni—N2—C10—C9	25.0 (2)
N2—Ni—N1—C9	−10.60 (14)	N1—C9—C10—N2	−31.2 (2)
O2—Ni—N1—C9	−115 (3)	C10—N2—C11—C12	−174.43 (19)
O1—Ni—N1—C9	168.43 (14)	Ni—N2—C11—C12	2.5 (3)
N1—Ni—N2—C11	174.22 (18)	N2—C11—C12—C18	3.5 (3)
O2—Ni—N2—C11	−7.22 (18)	N2—C11—C12—C13	−177.6 (2)
O1—Ni—N2—C11	71 (4)	C18—C12—C13—C14	−0.1 (3)
N1—Ni—N2—C10	−8.76 (14)	C11—C12—C13—C14	−179.0 (2)
O2—Ni—N2—C10	169.79 (14)	C12—C13—C14—C15	0.0 (3)
O1—Ni—N2—C10	−112 (4)	C13—C14—C15—C16	0.8 (3)
Ni—O1—C1—C7	−4.0 (3)	C17—O4—C16—C15	−3.2 (3)
Ni—O1—C1—C2	176.32 (13)	C17—O4—C16—C18	177.79 (16)
C3—O3—C2—C4	6.9 (3)	C14—C15—C16—O4	179.68 (19)
C3—O3—C2—C1	−173.86 (17)	C14—C15—C16—C18	−1.4 (3)
O1—C1—C2—O3	−0.4 (3)	Ni—O2—C18—C12	−6.0 (3)
C7—C1—C2—O3	179.85 (17)	Ni—O2—C18—C16	173.96 (13)
O1—C1—C2—C4	178.85 (18)	C13—C12—C18—O2	179.44 (19)
C7—C1—C2—C4	−0.9 (3)	C11—C12—C18—O2	−1.6 (3)
O3—C2—C4—C5	−179.16 (18)	C13—C12—C18—C16	−0.5 (3)
C1—C2—C4—C5	1.6 (3)	C11—C12—C18—C16	178.44 (18)
C2—C4—C5—C6	−0.7 (3)	O4—C16—C18—O2	0.3 (3)
C4—C5—C6—C7	−0.9 (3)	C15—C16—C18—O2	−178.73 (18)
C5—C6—C7—C1	1.6 (3)	O4—C16—C18—C12	−179.74 (17)
C5—C6—C7—C8	−175.9 (2)	C15—C16—C18—C12	1.2 (3)
O1—C1—C7—C6	179.6 (2)	C2S—N1S—C1S—O1S	179.27 (18)
C2—C1—C7—C6	−0.7 (3)	C3S—N1S—C1S—O1S	0.1 (3)
O1—C1—C7—C8	−3.0 (3)		

### Hydrogen-bond geometry (Å, °)

**Table:**

*D*—H···*A*	*D*—H	H···*A*	*D*···*A*	*D*—H···*A*
C4—H4A···O1S	0.95	2.62	3.310 (3)	130
C9—H9A···O2^i^	0.99	2.45	3.334 (3)	148
C10—H10A···O1^ii^	0.99	2.45	3.348 (3)	151

Symmetry codes: (i) −*x*+1, −*y*+1, −*z*+1; (ii) −*x*+2, −*y*+1, −*z*+1.

## References

[bb1] Abe, Y., Akao, H., Yoshida, Y., Takashima, H., Tanase, T., Mukai, H. & Ohta, K. (2006). *Inorg. Chim. Acta*, **359**, 3147–3155.

[bb2] Allen, F. H. (2002). *Acta Cryst.* B**58**, 380–388. 10.1107/s0108768102003890 12037359

[bb3] Assey, G., Gultneh, Y. & Butcher, R. J. (2010). *Acta Cryst.* E**66**, m654–m655. 10.1107/S1600536810017162 PMC2979541 21579300

[bb4] Feng, X., Du, Z.-X., Ye, B.-X. & Cui, F.-N. (2007). *Chin. J. Struct. Chem* **26**, 1033–1038.

[bb5] Miyamura, K., Mihara, A., Fujii, T., Gohshi, Y. & Ishii, Y. (1995). *J. Am. Chem. Soc.* **117**, 2377–2378.

[bb6] Oxford Diffraction (2009). *CrysAlis RED* and *CrysAlis PRO* Oxford Diffraction Ltd, Yarnton, England.

[bb7] Sheldrick, G. M. (2008). *Acta Cryst.* A**64**, 112–122. 10.1107/S0108767307043930 18156677

[bb8] Vasil’eva, S. V., Chepurnaya, I. A., Logvinov, S. A., Gaman’kov, P. V. & Timonov, A. M. (2003). *Russ. J. Electrochem.* **39**, 310–313.

